# Fatty Acid Profiling for the Authentication of Iberian Hams According to the Feeding Regime

**DOI:** 10.3390/foods9020149

**Published:** 2020-02-03

**Authors:** Raúl González-Domínguez, Ana Sayago, Ángeles Fernández-Recamales

**Affiliations:** 1 Department of Chemistry, Faculty of Experimental Sciences, University of Huelva, 21007 Huelva, Spain; ana.sayago@dqcm.uhu.es; 2 International Campus of Excellence CeiA3, University of Huelva, 21007 Huelva, Spain

**Keywords:** Iberian ham, authentication, feeding, fatty acids, oleic acid, artificial neural network

## Abstract

The quality and sensory characteristics of Iberian ham are closely related to the pig feeding regime. These are mainly due to the inclusion or not of acorns into the diet, which significantly increases the content of monounsaturated fatty acids in this food product. In this work, the fatty acid profile from subcutaneous fat samples was evaluated and modeled with various chemometric approaches as a potential tool for authentication of Iberian ham from three categories according to the rearing system: “Jamón de Bellota”, “Jamón de Cebo de Campo”, and “Jamón de Cebo”. The application of artificial neural networks provided satisfactory classification and prediction rates, with oleic acid being the most important variable driving this differentiation.

## 1. Introduction

The Mediterranean diet, mainly based on the consumption of vegetables, fresh fruits, cereals, fish and moderate quantities of wine, has been associated with reduced incidence of cerebral and cardiovascular diseases. The main source of fat in the traditional Mediterranean diet is olive oil, characterized by high contents of oleic acid, while the intake of meat should be limited. However, it should be noted that the Iberian pig, an autochthonous breed from southwest Spain, has lower content of saturated fatty acids and higher monounsaturated profile than other meats [1]. In this vein, it has been demonstrated that the moderate consumption of this meat elicits similar effects to those provoked by olive oil on the lipid profile and over cardiovascular risk factors [2,3]. Particularly, dry-cured hams are important sources of proteins, iron, phosphorous, B-vitamins, and other valuable nutrients, which determine the high nutritional value of this food product [4].

Iberian ham has a characteristic taste and flavor, highly appreciated by consumers over the world, which are mainly attributed to volatile compounds produced from lipid oxidation [5,6,7]. Multiple factors can influence the lipid composition of pig meat, thus significantly affecting the organoleptic quality of derived products, but it has been sufficiently proven that breed and the type of diet received by the pigs at the end of their fattening period before slaughtering are the main players [8,9]. The traditional exploitation system of Iberian pigs is called “Montanera”, an extensive feeding regime based on pasture and acorns over approximately the last three months of their lives. Acorns are rich in monounsaturated fatty acids [1], so the quality and market value of ham greatly depend on the inclusion or not of this fruit in the diet during these last three months. Thus, according to the type of feed, the Spanish legislation establishes three main categories for Iberian ham: “Jamón de Bellota”, from Iberian pigs exclusively fed with natural pasture and acorn, “Jamón de Cebo de Campo”, receiving mixed feeding with concentrate feeds and complemented with natural pasture and acorn, and “Jamón de Cebo”, exclusively fed with concentrate feeds [10].

Considering the great commercial value of Iberian ham and the crucial role of pig feeding on its final quality, numerous efforts have been made to develop sensitive analytical approaches for characterizing the chemical composition of this product. Most of the published literature is based on investigating the lipid profile of Iberian ham, particularly focusing on fatty acids, which have demonstrated a great potential to differentiate Iberian hams according to the feeding regime [11,12,13,14,15,16], but also by studying other components such as triacylglycerols [13,16] or the entire lipidomic profile [17,18]. In this context, the use of advanced chemometric tools to manage the complex data sets generated by using these analytical approaches is also of utmost importance. Various pattern recognition techniques have been frequently employed in food authenticity research, such as linear discriminant analysis, or partial least squares discriminant analysis [19,20], but the application of novel machine learning tools is gaining importance in recent years, including artificial neural network modeling, random forest, and support vector machines [21].

On this basis, the main aim of the present study was to investigate the fatty acid profile from subcutaneous fat as a potential authentication tool to discriminate Iberian hams according to the pig rearing system. To this end, the performance of various pattern recognition techniques, including linear discriminant analysis (LDA) and artificial neural network (ANN) modeling, was compared with the aim of obtaining suitable models for classification purposes. This work thus demonstrates that fatty acids can be potential descriptors for Iberian ham authentication and fraud detection, which is of great importance in the food industry due to the high commercial value of this product.

## 2. Materials and Methods 

### 2.1. Iberian Ham Samples

Sixty-three Iberian ham samples produced under the Protected Designation of Origin “Jamón de Huelva” were used in this study. Three study groups were considered: “Jamón de Bellota” (*N* = 25), “Jamón de Cebo de Campo” (*N* = 23), and “Jamón de Cebo” (*N* = 15). Samples of subcutaneous fat were collected before the dry-curing process by longitudinal cutting in the tail insertion area from the coxal region. The skin and muscle were removed, and the fat was minced and blended.

### 2.2. Lipid Extraction and Transesterification 

Lipid extraction and transesterification was performed according to the normative UNE-EN ISO 5508:1996. For lipid extraction, 10 g of subcutaneous fat was mixed with 10 mL of diethyl ether and homogenized during 10 min. Then, extracts were filtered through 0.45 µm filters and the solvent was removed by rotatory vacuum evaporation. For transesterification, 20 mg of the resulting solid residue was accurately weighed and dissolved with 4 mL of n-hexane. Afterwards, 200 µL of 2 M methanolic potassium hydroxide was added to the extract, and after mild shaking, the mixture was left to stand for 30 min until total clarification. The upper layer containing fatty acid methyl esters was finally transferred to injection vials.

### 2.3. Determination of Fatty Acid Methyl Esters

Fatty acid methyl esters (FAMEs) were analyzed by using a Hewlett-Packard 6890 Plus gas chromatograph equipped with split/splitless injector and flame ionization detector (FID). Separation was performed on a DB-23 capillary column (60 m × 0.25 mm i.d., 0.25 µm film thickness) coated with polar stationary phase (50% cyanopropyl, 50% methyl polysiloxane) (Agilent Technologies, Santa Clara, CA, USA). The GC conditions were as follows: oven temperature, 190 °C isothermal for 20 min; injector temperature, 250 °C; detector temperature, 275 °C; carrier gas, helium; carrier gas flow rate, 1.5 mL/min flow rate; flow split, 85:1; injection volume, 1.0 µL. Data were collected using the HP workstation, and fatty acid methyl esters were identified by comparing retention times with those from reference standard mixtures: lauric, myristic, palmitic, palmitoleic, margaric, heptadecenoic, stearic, oleic, linoleic, linolenic, arachidic, and gadoleic acids. The results were expressed as percentage of the total fatty acid methyl ester content determined in this study (i.e., sum of 12 FAMEs). Method repeatability was assessed by analyzing ten times the same extract, while reproducibility was estimated by analyzing ten extracts from the same subcutaneous tissue sample.

### 2.4. Data Analysis

Principal component analysis (PCA) and hierarchical cluster analysis (HCA) were first applied for preliminary exploration of data, and ANOVA was employed to find significant differences among the study groups. Then, linear discriminant analysis (LDA) and artificial neural network (ANN) were performed to build classification models. For supervised multivariate analysis (i.e., LDA and ANN), the dataset was randomly divided into two groups, a training set used to build the model, and a prediction set to test its performance. The percentage of samples taken for the prediction set was about 25%. To validate the recalling rate (effectiveness of classification in the training set) and the prediction ability (effectiveness of classification in the prediction set) of the methods applied, both training and prediction sets were repeated ten times for different constitutions. The average of hits obtained in the recalling and prediction from these ten runs was used as a measure of the classification performance. Regarding ANN modeling, several architectures were built by applying the multi-layer perceptron trained by the error back-propagation algorithm. In all cases, ANNs were two-layer neural networks with log-sigmoidal and bias transfer function, with three output neurons and as many input neurons as variables, and with a priori indeterminate number of hidden neurons. The output from the network was adjusted to a zero/one response. For back-propagation training, initial weights were taken randomly between −0.1 and 0.1, maximal epochs were 1000, and learning rate and momentum were fixed to 0.2 and 0.5, respectively, and were kept constant during training. The root-mean-squared (RMS) errors were computed to assess the effectiveness of the training. All statistical analyses were conducted using Statistica 8.0 software (StatSoft, Tulsa, OK, USA).

## 3. Results and Discussion

### 3.1. Fatty Acid Profiling of Iberian Hams

Following extraction and transesterification, the total fatty acid content from Iberian ham subcutaneous fat samples was profiled by using gas chromatography coupled to flame ionization detector (GC-FID). This method enabled the fast determination of 12 fatty acids with suitable analytical performance, as shown in Table 1. Repeatability and reproducibility, expressed as percentage of relative standard deviation (RSD), were below 0.2% and 0.4% for major fatty acids (percentage of total FAME content above 5%), while for minor species, these parameters were lower than 10%, in line with previous inter-laboratory studies [22]. 

Descriptive statistical analysis (i.e., mean, minimum, and maximum values, expressed as percentage of total FAME content) for these 12 fatty acids quantified in Iberian ham samples under study is also summarized in Table 1. Results were in good agreement with those reported by other authors for “Jamón de Huelva” samples [14,23], and for other Iberian ham samples (e.g., “Guijuelo”, “Dehesa de Extremadura”) [14]. As can be seen, the predominant fatty acids in all samples were oleic, palmitic, and stearic acids, which represented almost 85% of the total fatty acid content. Similarly to previous studies, total percentages of saturated (SFA), monounsaturated (MUFA), and polyunsaturated (PUFA) fatty acids were 33%, 57%, and 9%, respectively [14]. Interestingly, this higher proportion of MUFAs, coming from the intake of acorns during the pig fattening period [24], has been previously described as a characteristic feature of Iberian ham compared with dry-cured hams from white pigs [13] and pigs reared in intensive systems [25].

### 3.2. Characterization of Iberian Ham Fatty Acid Profiles According to the Feeding Regime

The fatty acid composition (expressed as mean ± standard deviation of the percentage of total FAME content) of subcutaneous fat from Iberian hams grouped according to the three fattening systems were subjected to ANOVA to look for significant differences (Table 2). “Jamón de Bellota” hams were characterized by higher percentage of oleic, gadoleic, and total content of monounsaturated fatty acids, while lauric, myristic, palmitic, palmitoleic, and stearic acids were increased in “Jamón de Cebo” samples, in agreement with previous studies [13,14,17,18]. The fatty acid concentrations in “Jamón de Cebo de Campo” ranged between the values observed in “Jamón de Bellota” and “Jamón de Cebo”, as expected since this feeding regime is based on mixed feeding with concentrate feeds and natural pasture and acorns. Proportions of polyunsaturated fatty acids were similar in the three feeding systems considered, in agreement with previous studies [1,11].

**Figure 1:**
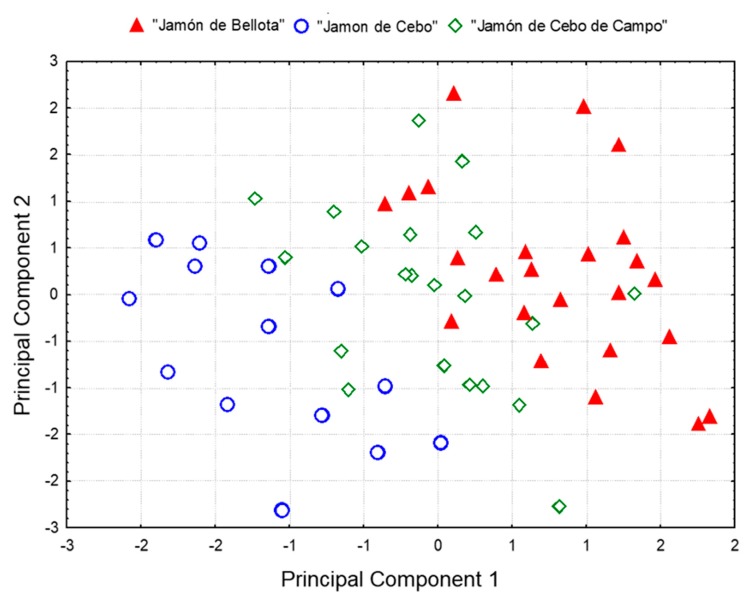
Principal component analysis (PCA) scores plot showing the distribution of samples from the three study groups in the plane defined by the two first principal components.

Complementarily, principal component analysis (PCA), and hierarchical cluster analysis (HCA) were also applied for a preliminary data exploration and for visualizing data trends. When PCA was applied, four principal components with eigenvalues higher than 1.0 were extracted (Kaiser criterion), accounting for 87.2% of the total variance. Three overlapped clusters can be observed in the two-dimensional scores plot defined by the two first principal components (Figure 1), with “Jamón de Bellota” samples situated on the left side of the plot, “Jamón de Cebo” samples characterized by positive scores on PC1 (39.78% of total variability explained), and “Jamón de Cebo de Campo” samples clustered between these two groups. Variables contributing to this clustering (loading values higher than 0.7) were oleic acid (negative values in PC1), lauric, myristic, palmitic, and palmitoleic acids (positive values in PC1). Only two variables presented significant loadings on PC2 (19.44% of total variability explained): arachidic and gadoleic acids. This grouping of samples along PC1 clearly reflects the effect of diet on the fatty acid composition of subcutaneous fat, from higher proportions of oleic acid in “Jamón de Bellota” as a result of feeding with acorns, to increased content of SFA in “Jamón de Cebo” [26].

**Figure 2:**
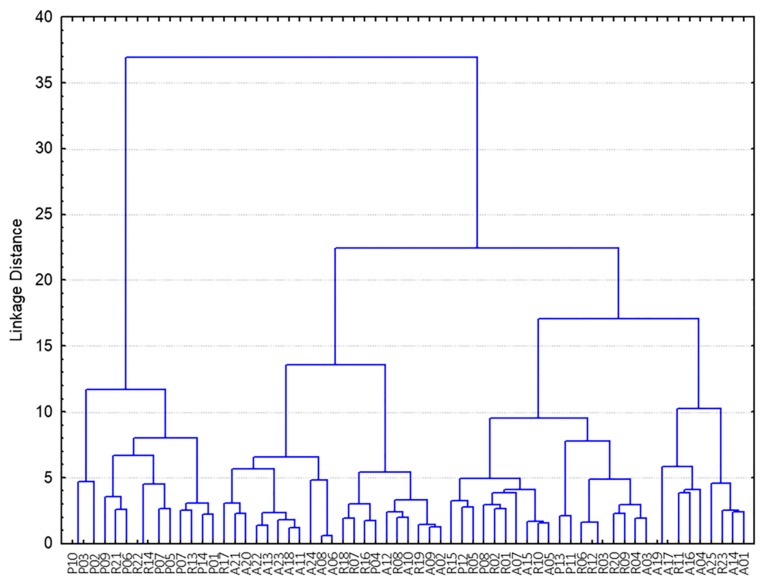
Hierarchical cluster analysis (HCA) dendrogram showing the distribution of samples from the three study groups. P: “Jamón de Cebo”, R: “Jamón de Cebo de Campo”, A: “Jamón de Bellota”.

Similar results were observed when HCA (Ward’s method of agglomeration and Euclidean distances) was applied to the same dataset for evaluating similarity between samples. Although the composition of the different clusters is not so evident, three main groups can be identified in the dendrogram represented in Figure 2. One first group contained nearly all “Jamón de Cebo” and some “Jamón de Cebo de Campo” samples, while a second group was mainly composed by “Jamón de Bellota” samples (14 “Jamón de Bellota”, 6 “Jamón de Cebo de Campo”, 1 “Jamón de Cebo”). The third cluster was more heterogeneous, containing both “Jamón de Cebo de Campo” (13) and “Jamón de Bellota” (11), as well as some “Jamón de Cebo” samples (4).

### 3.3. Classification Models for Iberian Ham Authentication

Multiple supervised pattern recognition procedures and machine learning algorithms have been recently proposed in food research to solve authentication problems for various foods with high commercial value, such as olive oil [27,28,29], strawberry [30,31,32], or wine [33,34]. In this study, two statistical multivariate classification methods were tested with the aim of building classification models for discriminating Iberian ham varieties. Linear discriminant analysis (LDA) was first employed to obtain suitable classification rules for assigning categories to samples, being oleic and margaric acids the most discriminant variables. A correct global classification of 96% was obtained by using the training set (“Jamón de Bellota” *N* = 15, “Jamón de Cebo de Campo” *N* = 15, “Jamón de Cebo” *N* = 10), which was successfully validated in the prediction set (89.2% success rate). All study groups showed good percentages of correctly classified cases (Table 3). 

Finally, artificial neural network (ANN) modeling was also applied for classifying Iberian hams, for which different neural network structures, or multilayer perceptrons (MLP), were compared. As a first step, three-layer networks with different number of nodes in the hidden layer (12 × 3 × 3, 12 × 4 × 3, 12 × 8 × 3) and four-layer network with two nodes in each of the two hidden layers (12 × 2 × 2 × 3) were employed. Previous results from PCA were also considered to reduce the number of inputs into the MLP and take into account possible correlation among variables, so that the four main principal components were used as inputs (4 × 8 × 7 × 3). Furthermore, two additional MLPs were also built by using the seven most discriminant variables in the PCA as inputs (7 × 2 × 2 × 3) or only the most discriminant one (1 × 4 × 3). Table 3 shows the percentage of correct classification and prediction for all these networks, which gives satisfactory classifications with maximum errors of 15%. It should be noted that several of these models yielded 100% classification and prediction rates for the three study groups, clearly surpassing the performance provided by LDA, probably due to the intrinsically non-linear nature of the class distribution. To assess the effectiveness of the training by applying the back propagation method, RMS errors between the actual and desired network outputs are listed in Table 4 for the training, cross-validation, and tests sets for all these models. Interestingly, the best results were obtained by applying the 1 × 4 × 3 MLP structure, in line with findings from LDA.

## 4. Conclusions

To sum up, it can be concluded that the fatty acid profile of subcutaneous fat is strongly influenced by the feeding regime, with oleic acid being the most important variable driving this differentiation. This therefore represents a first step towards the development of a suitable tool for Iberian ham authentication. In this work, we demonstrated the need of applying advanced chemometric tools to efficiently determine the pig rearing system. The combination of classical multivariate statistical models and artificial neural network provided good performance for pattern recognition and classification. These results could be a starting point for the implementation of quality control assays in the food industry for fraud detection and authentication of Iberian hams.

## Figures and Tables

**Table 1:** Analytical performance of the gas chromatography-flame ionization detector (GC-FID) method in terms of repeatability and reproducibility. Mean, minimum, and maximum concentrations (expressed as percentage of total fatty acid methyl ester, FAME, content) found in Iberian ham samples under study.

Fatty Acids	Retention Time	Repeatability (RSD, %)	Reproducibility (RSD, %)	Mean	Min.	Max.
Lauric acid (C12:0)	3.63	8.7	9.3	0.07	0.05	0.08
Myristic acid (C14:0)	4.28	1.1	1.4	1.32	1.11	1.51
Palmitic acid (C16:0)	5.52	0.1	0.1	21.39	19.53	23.57
Margaric acid (C17:0)	5.73	1.0	0.8	0.32	0.25	0.45
Stearic acid (C18:0)	6.41	3.3	5.2	10.07	7.90	12.19
Arachidic acid (C20:0)	6.78	2.9	5.6	0.18	0.14	0.23
Palmitoleic acid (C16:1*n*-7)	7.71	0.2	0.35	2.23	1.70	2.94
Heptadecenoic acid (C17:1*n*-7)	8.25	0.04	0.09	0.32	0.24	0.43
Oleic acid (C18:1*n*-9)	9.02	0.1	0.4	53.29	49.97	56.40
Gadoleic acid (C20:1*n*-11)	10.21	2.2	2.6	1.55	1.22	2.00
Linoleic acid (C18:2*n*-6)	11.55	3.2	6.3	8.74	6.99	11.29
Linolenic acid (C18:3*n*-3)	12.32	0.5	1.9	0.51	0.36	0.86

Min., minimum; Max., maximum.

**Table 2:** Fatty acid composition of subcutaneous fat from Iberian hams (expressed as mean ± standard deviation of the percentage of total FAME content).

	“Jamón de Bellota”	“Jamón de Cebo ”	“Jamón de Cebo de Campo”
C12:0	0.064 ± 0.004 ^a^	0.071 ± 0.004 ^b^	0.067 ± 0.005 ^a^
C14:0	1.27 ± 0.07 ^a^	1.40 ± 0.08 ^b^	1.33 ± 0.08 ^c^
C16:0	20.52 ± 0.62 ^a^	22.66 ± 0.67 ^b^	21.51 ± 0.70 ^c^
C17:0	0.31 ± 0.03	0.33 ± 0.06	0.31 ± 0.04
C18:0	9.52 ± 0.58 ^a^	10.87 ± 0.67 ^b^	10.09 ± 0.57 ^c^
C20:0	0.18 ± 0.02	0.19 ± 0.02	0.19 ± 0.02
Total saturated	31.87 ± 1.05 ^a^	35.61 ± 1.13 ^b^	33.50 ± 1.06 ^c^
C16:1*n*-7	2.09 ± 0.28 ^a^	2.38 ± 0.30 ^b^	2.28 ± 0.31 ^ab^
C17:1*n*-7	0.32 ± 0.05	0.34 ± 0.06	0.32 ± 0.05
C18:1*n*-9	54.80 ± 0.65 ^a^	50.96 ± 0.69 ^b^	53.18 ± 0.64 ^c^
C20:1-11	1.63 ± 0.16 ^a^	1.44 ± 0.14 ^b^	1.54 ± 0.15 ^ab^
Total monounsaturated	58.83 ± 0.85 ^a^	55.12 ± 0.69^b^	57.31 ± 0.68 ^c^
C18:2*n*-6	8.77 ± 0.85	8.80 ± 1.01	8.68 ± 0.97
C18:3*n*-3	0.53 ± 0.10	0.47 ± 0.09	0.51 ± 0.06
Total polyunsaturated	9.30 ± 0.90	9.27 ± 1.05	9.91 ± 1.02

Superscript letters within each row indicate significant differences between groups sharing the same letter according to Tukey HSD test (*p* < 0.05).

**Table 3:** Statistical performance for linear discriminant analysis (LDA) and artificial neural network (ANN) classification methods.

	LDA	ANN						
		MLP 12 × 3 × 3	MLP 12 × 4 × 3	MLP 12 × 8 × 3	MLP 12 × 2 × 2 × 3	MLP 4 × 8 × 7 × 3	MLP 7 × 2 × 2 × 3	MLP 1 × 4 × 3
**Classification Rate (%)**								
“Jamón de Bellota”	96	100	100	93.3	100	93.3	100	100
“Jamón de Cebo”	100	100	100	100	100	100	100	100
“Jamón de Cebo de Campo”	93.9	100	92.3	93.3	100	100	100	100
**Prediction Rate (%)**								
“Jamón de Bellota”	96	100	100	75	100	100	66.7	100
“Jamón de Cebo”	96	100	100	100	100	100	100	100
“Jamón de Cebo de Campo”	76.8	100	75	100	100	66.7	100	100

MLP, multilayer perceptron.

**Table 4:** Calculated root-mean-squared (RSM) errors for the seven multilayer perceptron (MLP) architectures compared in the study.

	MLP 12 × 3 × 3	MLP 12 × 4 × 3	MLP 12 × 8 × 3	MLP 12 × 2 × 2 × 3	MLP 1 × 4 × 3	MLP 7 × 2 × 2 × 3	MLP 4 × 8 × 7 × 3
Training set	0.063	0.133	0.015	0.036	0.001	0.045	0.170
Verification set	0.085	0.169	0.448	0.087	0.018	0.231	0.286
Test set	0.115	0.3678	0.443	0.481	0.091	0.112	0.352
